# Evidence-Based Eating Patterns and Behavior Changes to Limit Excessive Gestational Weight Gain: A Scoping Review

**DOI:** 10.3390/ijerph21010015

**Published:** 2023-12-21

**Authors:** Kiley Ketchum, Cecilia M. Jevitt

**Affiliations:** Midwifery Program, Department of Family Practice, Faculty of Medicine, University of British Columbia, Vancouver, BC V6T 1Z3, Canada; kketch16@student.ubc.ca

**Keywords:** pregnancy, gestational weight gain, nutrition, behavior, weight gain, obesity, Mediterranean diet, low-glycemic index diet, DASH diet, mindful eating, intuitive eating, meal planning, portion control

## Abstract

Background: International prenatal care guidelines set a standard for clinicians to discuss gestational weight gain with their patients along with the complications associated with prepregnancy obesity and excessive gestational weight gain. Clinicians often lack evidence-based eating, nutrition, and activity strategies to share with patients. Methods: This systematic review aimed to find eating patterns and behaviors that could be used safely during pregnancy to limit excessive gestational weight gain. PubMed, MEDLINE, and Web of Science were searched for research or systematic reviews performed in the United States or Canada and published in English from 2013 to 2023. Keyword search terms included weight, manage, behavior, strategy, strategies, gestational weight gain, and nutrition. Excluded research used pediatric or adolescent populations, restrictive diets, such as no carbohydrate or no fat diets, fasting, bariatric surgery, weight loss medications, private industry or profit-earning programs using food brands, or specific diet programs. Results: A total of 844 abstracts were retrieved, with 103 full-text studies reviewed. Behaviors had to be useful for maintaining a healthy gestational weight gain and had to be safe for use during pregnancy. Behaviors useful during pregnancy included meal planning, home meal preparation, portion control, using diets such as the Mediterranean diet, the low-glycemic index diet, and the Dietary Approaches to Stop Hypertension diet (DASH), regular physical activity, sleeping 6–7 h a night, mindful eating, intuitive eating, and regular seif-weighing. Conclusion: The evidence-based strategies outlined in this review are safe for use during pregnancy and can assist patients in avoiding excessive gestational weight gain while maintaining the nutrition needed for healthy fetal growth.

## 1. Introduction

Excessive gestational weight gain has been linked with gestational diabetes, hypertensive disorders of pregnancy, including preeclampsia, prolonged labor, fetal macrosomia, maternal postpartum weight retention, future obesity, and diabetes and cardiac disease for the mother and newborn for decades [1,2,3,4,5,6,7], yet the most effective counseling and behaviors to reduce excessive gestational weight gain remain disputed and poorly researched. Despite growing controversy over the use of body mass index (BMI = kg/m^2^) as a measurement in health care, the 2009 U.S. Institute of Medicine Pregnancy BMI-based Weight Gain Guidelines remain the international standard for research and clinical care in gestational weight gain [1,2,3,4]. 

Obesity (BMI ≥ 30) is one of the most common conditions to potentially complicate pregnancy in North America, with more than 30% of individuals in some locations entering pregnancy with obesity [1,2,3,4,7]. Individuals with obesity are more likely to gain weight excessively during pregnancy and retain weight in the postpartum period [1,2,3]. Obesity and excessive weight gain individually increase the risk for metabolic and cardiac disease in pregnancy and have a synergistic effect to further increase perinatal risk [1,3]. Optimal prenatal weight gain occurs within ranges defined by the USA Institute of Medicine guidelines [1,2,3,4]. For individuals with overweight and obesity, the weight gain targets are constrained to 7–11.5 kg (15–25 pounds) and 5–9 kg (11–20 pounds), respectively. These ranges are contrasted with the target weight gain for individuals with normal prepregnancy BMIs, which is 11.5–16 kg (25–35 pounds). The 11.5 to 16 kg weight gain range is what most women consider a normal pregnancy weight gain. The need to gain weight during pregnancy for the health and growth of the fetus while avoiding excess weight gain poses a unique challenge for individuals with overweight and obesity [1,2,4].

Routine weighing was a traditional component of prenatal care visits, stemming from eras of inadequate food supply and surveillance for inadequate weight gain and preeclampsia-related fluid retention. In the mid-1990s, prenatal care providers in many English-language countries abandoned discussions about gestational weight gain and routine prenatal weighing to limit fat-shaming and weight bias during prenatal care [8]. Additionally, perinatal care providers were reluctant to discuss weight management, fearing that individuals would be offended or experience weight stigma [3,8]. The Society of Obstetricians and Gynecologists of Canada (SOGC) [3], the Academy of Nutrition and Dietetics [4], and ACOG [1] have position statements urging clinicians to review the risks associated with obesity and excessive gestational weight gain as a part of prenatal informed consent discussions. Given the effect of excessive gestational weight gain and obesity on not only the pregnancy but also on the future health of the mother and newborn, evidence-based weight management counseling is an ethical imperative [4].

Seeking to understand what weight management behaviors individuals have used in the past can be the first step in counseling about gestational weight gain strategies [9,10]. Effective weight management strategies must be separated from behaviors that cause weight loss in the short term and can be injurious to prenatal health, such as fasting or the use of appetite suppressant drugs [11]. This scoping review sought evidence-based eating patterns and weight management behaviors that could be suggested by antenatal care providers to assist individuals in preventing excessive gestational weight gain.

## 2. Materials and Methods

In order to find evidence-based eating patterns and weight management strategies, a scoping review was conducted in the following databases: PubMed, MEDLINE, and Web of Science. Keyword search terms included weight, manage, behavior, strategy, strategies, gestational weight gain, and nutrition. Culture influences eating and activity behaviors, and industrial food processing and distribution impacts the foods available to individuals; therefore, the selected research was limited to studies that occurred in Canada or the United States. Research was limited to that published in English from 2013 to 2023. Given the multiple factors that influence individual eating patterns, research and professional guidelines from multiple health disciplines were additionally searched. Professional guidelines from Canada and the United States for weight management and gestational weight gain were reviewed; however, many of these guidelines are available by subscription only. Therefore, subscription-only international guidelines were cited, and then original research supporting the guideline conclusions was included in the review. 

**Figure 1:**
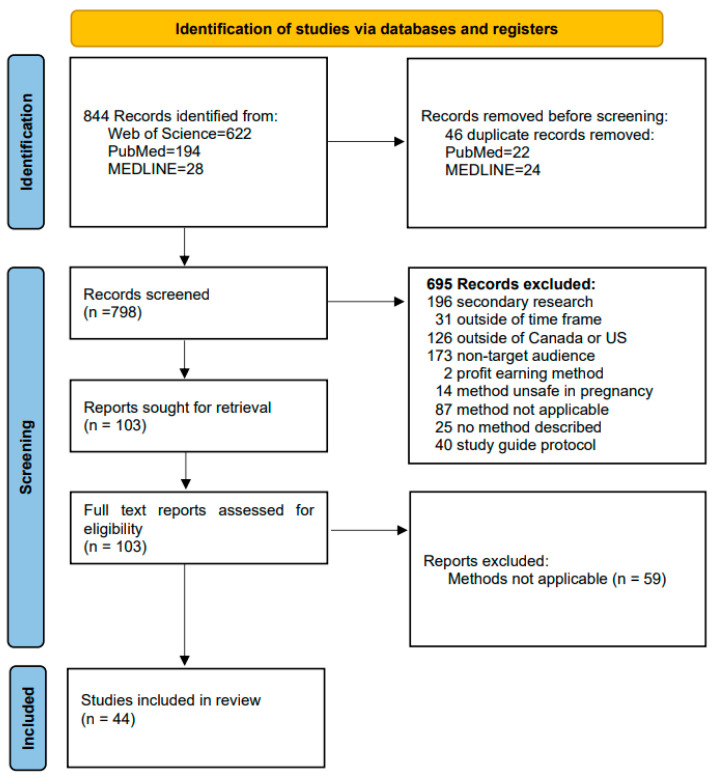
PRISMA diagram evidence-based behavior changes and gestational weight gain.

Studies manipulating diet and behaviors during pregnancy are scarce compared to research performed outside of pregnancy; therefore, studies performed outside of pregnancy were included but had to include weight maintenance strategies that were safe during pregnancy. Studies including restrictive diets such as no carbohydrate or no fat diets, fasting, bariatric surgery, weight loss medications, and herbs were excluded. Private industry or profit-earning programs using food brands or specific diet programs were also excluded. Because the research was intended for use during pregnancy, studies using pediatric populations were excluded. Studies using technological interventions such as reminders sent to mobile phones were excluded because up to 30% of families in the United States have poor internet or mobile phone connectivity [12], although Canada claims 93.5% of families have high-speed internet access [13]. Due to the numerous manuscripts published related to eating patterns, weight management strategies, and gestational weight gain, the inclusion of meta-analyses and synthetic reviews was favored (Figure 1). Full-text reviews were performed by two reviewers on 103 manuscripts with 44 studies forming the evidence base (Appendix A).

## 3. Results

Individuals have limited knowledge about healthy gestational weight gain. In one USA study of 330 pregnant individuals, almost one-half of women (42.8%) planned to gain weight that was above the recommended amount because of incorrect knowledge about prenatal weight gain. Almost one-in-five planned to gain below the recommended amounts (18.3%) [14]. Women who gained excess weight were more likely to have planned to do so based on erroneous weight gain knowledge [14]. The SOGC recommends individualized gestational weight gain goals that are revised as pregnancy advances [3].

Two researchers reviewed 44 studies that form the core of this review with 10 randomized controlled trials, 8 prospective cohort studies, 4 longitudinal observation studies, and 4 retrospective reviews being the primary sources of data. Additionally, six national guidelines containing recommendations for gestational weight gain were reviewed and primary studies used in these guidelines were reviewed.

### 3.1. The Eating Environment

Eating is more complex than energy intake. Eating is often a social behavior and is linked with celebration. Eating is integrated into usual social waking and work schedules; therefore, mealtimes may affect total intake. Proximity, accessibility, and visibility of food are positively associated with food intake [5,15,16]. In one study of 198 low-income pregnant women, food security was associated with increased fruits and vegetables available in the home and their subsequent increased intake by women [15]. Another study of 209 participants who were predominantly female (83%) examined the availability of fruits and vegetables in the home, meal planning, and using a shopping list, finding that none of those measures was significantly associated with weight loss [16]. In women, eating frequency has not been shown to independently influence energy intake or anthropometric outcomes. The Dieticians of Canada [6] found that there is inadequate quality evidence to recommend for or against regularly consuming breakfast for the purpose of weight management; however, one recent meta-analysis of 44 observational studies found that skipping breakfast increased the risk of overweight and obesity with an odds ratio of 1.47 (95% CI 1.139–1.57; *p* = 0.002) for those with overweight and obesity [17]. Instead of meal skipping, some patients propose grazing as a weight management strategy. One study found that frequent grazing that starts and ends relatively late in the day was associated with higher energy intake and decreased dietary quality [18]. This increased consumption of discretionary foods was associated with increased BMI and waist circumference [18]. Intentional or planned meals at conventional times seemed to yield lower overall calorie intake when compared to grazing or more than three meals per day. Planning meals and snacks is recommended for prenatal nutrition by the Society of Obstetricians and Gynecologists of Canada [3]. 

### 3.2. Energy Intake

Many cultural changes have influenced food availability and meal portions in the last 50 years, including improved economies, urbanization, and increased technology in food processing [19]. Adults with obesity tend to underestimate their portion sizes when compared to those of normal weight [5,20]. Decreasing portion size, a frequently recommended strategy, has been linked to decreased energy intake without increased ratings of hunger. One randomized controlled study of 53 women demonstrated that providing to-go containers at the start of a large meal decreased consumption during the meal, thereby reducing caloric intake [21]. Portion controlled meals may help to reduce portion sizes but have not been shown to be sustainable over time [22]. 

Food reward has been defined as an eating behavior where food has momentary value to the individual, and the pleasure associated with ingestion drives the eating [23]. Women with obesity who view food as a reward may view larger portion sizes as normal, when compared to those of normal weight [5]. Larger portions at one meal have not been shown to decrease caloric intake at a later time to compensate for the large intake, and larger portions are associated with increased energy intake [5,21,24]. Women may need education in portion size to gain the weight needed for optimal fetal nutrition and growth without adding excess weight. 

### 3.3. Types of Intake and Quality

Randomized controlled trials (RCT) indicate that increasing vegetable and fruit intake is associated with weight loss and maintenance of loss when combined with other dietary weight loss interventions [5]; however, data from epidemiological studies are inconsistent, with some studies reporting greater vegetable and fruit intakes to be associated with lower body weight, greater weight, or no difference in body weights [5,15,16]. A 2020 systematic review by the United States Department of Agriculture found that intake patterns that emphasized fruits, vegetables, legumes, and whole grains while reducing alcohol, red meats, and sugar-sweetened foods and drinks were associated with lower BMIs or percent body fat [25]. Planning meal and snack behaviors and associations with fruits and vegetables in the home were nonsignificant in one study (*p* > 0.05) [15,16]. The plant-based diets that are described in this section are contrasted with what has been labeled the Western diet [26,27]. The Western diet is characterized by frequent servings of red meats and highly processed meats with sugar and salt added as flavor enhancers. These highly processed foods are carbohydrate and calorie-dense and often include saturated fats, refined grains and sugars, salt, and artificial colors and preservatives [26,27]. Western diet foods have some economic and social benefits when compared to fresh fruits and vegetables. Processed foods can be made from inexpensive carbohydrates and fats, can be packaged for easy distribution, and have long shelf lives [7]; however, high intake of these foods’ limits both the micronutrients (vitamins and minerals) and macronutrients (polyunsaturated and monounsaturated fats and proteins) necessary for healthy fetal development [26,27].

In one RCT, the authors of the Obesity Canada Clinical Practice Guidelines found that a Mediterranean diet with no weight loss focus did not have much effect on body weight or waist circumference despite its positive effect on glycemic control and cardiovascular risk factors [28,29]. The Obesity Canada Clinical Practice Guidelines recommend that many diets, including the Dietary Approaches to Stop Hypertension diet (DASH diet), the Mediterranean diet (MedDiet), and low-glycemic index diets, do not cause weight loss without calorie or portion restriction; however, all improve metabolic parameters, including lipids and blood glucose, and are associated with lower levels of diabetes and cardiac disease [28,29]. A 2015 literature review attempted to define the MedDiet [30]. Definitions consistently included recommendations for high intake of vegetables with emphasis on leafy greens, fruits, whole grain options, nuts, and pulses/legumes in addition to portion-controlled healthy fat sources, such as extra virgin olive oil. Intakes of fish, other meat, and dairy products were to be moderate, with low intakes of sugared foods. A 2020 systematic review and meta-analysis of 80 meta-analyses, including 485 unique RCTs, found suggestive evidence that the MedDiet, the DASH diet, and vegetarian dietary patterns decreased body weight and BMI [31]. The DASH diet is similar to the MedDiet and consists of an intake base of vegetables, fruits, and whole grains, fat-free or low-fat dairy products, fish, poultry, beans, nuts, and vegetable oils. Foods that are high in saturated fat, such as fatty meats, full-fat dairy products, and tropical oils, such as coconut, palm kernel, and palm oils are limited in the DASH diet as are sugar-sweetened beverages and sugared foods [32]. 

A low-glycemic index (GI) diet appears to be as effective as other modestly effective diets in reducing body weight and body fat in adults with higher weights (BMI ≥ 25 kg/m^2^) [6]. A 2019 systematic review and meta-analysis of 101 RCTs (n = 8527) concluded that adults with a BMI >25 kg/m^2^ who follow a low-GI diet can see modest decreases in body weight and body fat that are in line with the outcomes seen in other diets. This effect was only present in individuals with normal glucose tolerance, while individuals with impaired glucose tolerance or type 2 diabetes did not decrease body weight or body fat in response to a low-GI diet [33]. The 2020 Obesity Canada guidelines for obesity management in adults recommend that a low-GI diet can be used to reduce body weight and improve cardiometabolic markers (glycemic control, blood lipids, blood pressure) in adults with obesity. This recommendation was based on the results of a systematic review that defined overweight and obesity by combining BMI classifications (≥25 kg/m^2^) [34,35].

Dairy consumption leads to weight loss, decreased body fat, and decreased waist circumference when it is included in the context of an energy-restricted diet [6]; however, weight loss is discouraged during pregnancy. Body weight increased with dairy consumption when energy was not restricted; body fat and waist circumference (WC) were not affected [6]. A 2019 meta-analysis of RCTs assessed the effect of increasing daily food intake to increase calcium intake. Increasing dairy intake to approximately 3 servings daily (approximately 1300 mg of Ca/d) was not shown to be an effective weight reduction strategy in adults. There was, however, an indication that approximately 3 servings of dairy may facilitate fat loss on weight reduction diets in the short term [36]. Table 1 synthesizes recommended foods and servings from diets that are healthy during pregnancy [26,27,28,29,30,31,32,33,34,35]. Clinicians can use these foods and portions when advising patients about healthy eating. 

#### Probiotics

Probiotics have been shown to decrease body weight, BMI, and body fat percentage compared to placebo in adults with BMI ≥ 25 kg/m^2^, although the changes are small and may not be clinically important [6]. One 2021 systematic review of the use of probiotics and synbiotics in RCTs of weight loss in people with overweight and obesity found that the intake of probiotics or synbiotics could lead to significant weight reductions, either by maintaining habitual lifestyles or in combination with energy restriction and/or increased physical activity for an average of 12 weeks [37]. Specific strains belonging to the genus Lactobacillus and Bifidobacterium were used most often and showed the best results in reducing body weight. The optimal dose and type of probiotic for weight management is not known. Primarily, research regarding probiotic use during pregnancy is sparse; therefore, probiotic use for weight management during pregnancy cannot be advised.

### 3.4. Activity 

#### 3.4.1. Physical Activity

Exercise reduces the risk of common pregnancy complications [38], such as low back pain, and has been shown to lower the incidence of excessive gestational weight gain, gestational diabetes, gestational hypertension, preterm birth, low birth weight, and cesarean birth [39]. These benefits directly impact the risks associated with elevated BMI in pregnancy [3]. A 2017 meta-analysis of randomized trials on diet and physical activity-based interventions in pregnancy included 36 trials with 12,526 women [40] and concluded that an antenatal diet and physical activity-based interventions reduced gestational weight gain and lowered the odds of caesarean section.

The SOGC recommends reducing sedentary behavior in pregnancy [3] and further advises that pregnant people should do 150 min of moderate-intensity activity each week, over a minimum of 3 days per week [38]. Supervised physical activity (PA) programs or personalized prescriptions and goals improve adherence to gestational weight gain targets [3]. The physical activity does not have to be high intensity. A yoga intervention improved long-term weight loss in comparison to the control group in one study [41]. Weight loss maintainers engaged in more moderate-vigorous activity in bouts greater than 60 min when compared to controls with and without obesity [42]. There was no evidence for an association between the volume of moderate-to-vigorous–intensity exercise and weight regain across 12 months following clinically relevant weight loss [42]. Exercise volumes lower than those currently recommended for weight loss maintenance, when completed in conjunction with a behavioral weight-maintenance intervention, may help reduce excessive gestational weight gain and minimize weight regain over 12 months postpartum [43].

#### 3.4.2. Sleep

Adequate sleep patterns have been shown to be associated with improved gestational weight gain [44]. A 2014 systematic review found 18 studies where sleep was manipulated, and weight was measured. The available experimental literature suggested that sleep restriction increases food intake, but the resulting weight change was variable [44]. A later meta-analysis of 153 prospective cohort studies with a cumulative total of 5,172,710 participants examined the relationship between short sleep duration and obesity. Included studies had follow-ups of at least one year. Inadequate sleep increased the risk of obesity (RR1.38, 95% CI 1.25–1.53) [45].

### 3.5. Behavior Changes

Self-efficacy enhances many health-promoting behaviors, including weight management [46]. Patients have varying levels of self-efficacy and may need additional support from providers to optimize gestational weight gain. Clinicians may guide patients toward the pursuit of intrinsic goals and autonomous motivation for the regulation of eating [19]. Motivational interviewing, a patient-centered counseling style, benefits weight management counseling and goal setting [47]. Health professionals can engage in supportive behaviors by considering their patients’ points of view, by helping individuals identify barriers and obstacles toward healthy eating and activity, and by providing constructive feedback (competence support) [19]. Personalized weight management strategies and simple messages to reinforce those strategies increase weight management success [39]. As an example, one program targeted weight gain optimization through dietary advice, increasing physical activity, and stress reduction techniques supported with routine phone sessions discussing those behavioral strategies for gestational weight gain. Participants had prepregnancy BMIs from 25 to 39. Those in the intervention group had a reduced weekly rate of weight gain compared to those in the control group; however, there were no differences in perinatal complications, including gestational diabetes, between the two groups [48].

Mindful and intuitive eating have been proposed as methods for regulating intake and managing weight. Mindfulness has been defined as a “temporary state of non-judgment, non-reactive, present-centered attention and awareness that is cultivated during meditation” [49]. Clinicians have coached patients to use mindfulness techniques to develop an awareness of hunger and satiety cues, emotional states associated with eating, and external triggers to eat when not hungry [49]. Non-pregnant women with overweight and obesity were taught an intervention that was designed to improve the following three behaviors: stress-related eating, mindless eating, and dietary compliance with the DASH diet. Mindfulness eating quality scores improved significantly (*p* = 0.001), and stress-related eating scores improved (*p* = < 0.001) along with body weight (*p* = 0.02). Mindful eating may be effective in addressing stress-related eating [49]. In a small study with 24 participants, there was insignificantly decreased energy intake with mindful eating compared to energy intake when eating normally [50]. In one study of 16 participants, half of whom had obesity, higher energy intake was associated with a greater number of bites, reduced eating speed, and higher BMI. A higher percentage of meals within the slowest eating episodes (80%) were consumed during screen time (i.e., watching TV, laptop, or phone) compared with the fastest eating episodes (30%) [51]. Mindful strategies may contribute to weight regulation over prolonged periods [50].

Intuitive eating is a method of eating that relies on physiologic hunger cues and satiety signals instead of prescribed diets or portion control [52]. Intuitive eating has not been extensively studied but one study drew data from Project EAT-III, a population-based study of 2287 male and female young adults (mean age: 25.3 years) [53]. Project EAT-III investigated intuitive eating behaviors. Intuitive eating was inversely associated with BMI in both genders. Those who reported trusting their body to tell them how much to eat had lower odds of disordered eating behaviors compared to those that did not have this trust. Females who stopped eating when they felt full had lower odds of chronic dieting and binge eating than those who do not stop eating when full [53].

Limiting screen time during eating is often linked with mindful and intuitive eating. Screen watching while eating is hypothesized to be less intentional than eating without screen entertainment or work. Screen watching may draw attention away from food enjoyment, including aroma and taste, and may inhibit the sensing of satiety signals, the basis of intuitive eating. No studies were found that demonstrated a link between screen time and excess weight in adults. May studies of children recommend limiting screen time during eating; however, extended screen time may be a proxy for reduced physical activity and its effect on weight [40]. 

Monitoring of gestational weight gain is recommended by the SOGC and ACOG with the SOGC further recommending self-weighing during pregnancy [1,3]. In one small study with 30 participants, half were randomized to usual prenatal care and half were randomized to an educational intervention with weekly counseling by a registered dietitian with weekly weighing. Women remaining within weight gain goals continued with education while women exceeding goals received more intensive education and counseling. Those in the intervention group had 21% lower gestational weight gain than the control group [53]. Attention to weight gain in prenatal care must be preceded by a thorough history that excludes prior or current eating disorders.

## 4. Discussion

Optimizing gestational weight gain through behavior changes is known to have health benefits for mothers and newborns. Pregnant patients want this education [54,55]. Nutrition and weight gain advice are traditional components in prenatal care, although, recently, some clinicians have linked this with weight shaming and the potential triggering of anxiety and eating disorders [56,57,58]. Avoidance of nutrition and weight management education in antenatal care may indicate health care provider discomfort with these topics or lack of knowledge [56]. Screening for eating disorders is a routine part of antenatal care. As with many co-morbidities, individuals with eating disorders or metabolic diseases are referred to specialized care team members. Weight conversations are not reserved for those with BMIs over 25. The strategies in Table 1 are not weight loss-focused; they promote adequate nutrition to avoid malnutrition without excessive weight gain. 

Pregnancy is a time of vulnerability when those with a history of an eating disorder may feel out of control as their body image changes with fetal growth and gestational weight gain. Udo and Grilo’s epidemiological analysis estimated the lifetime and 12-month prevalence of anorexia nervosa, bulimia nervosa, and binge eating disorder as follows: 0.80 and 0.05%, 0.28 and 0.14%, and 0.85 and 0.44%, respectively, based on diagnostic criteria from DSM-IV to DSM-5 [59]. These USA prevalence statistics include males and females and are not pregnancy-specific. Canadian prevalence for eating disorders is similar [60]. Antenatal care providers should feel competent in providing equitable care to people with overweight and obesity. Obesity is noted as a risk factor in antenatal care. As with all risk factors, it is integral that individualized care is available to patients. When clinicians are aware of their biases, proceed with a trauma-informed approach, and provide tailored referrals where a disordered eating history is present, patients have access to equitable care. All people accessing antenatal care should be asked permission to discuss weight or GWG regardless of ED history. Additionally, patients should be asked for permission for a weight measurement instead of this being considered routine. If weighing is anxiety-provoking, the clinician can discuss weighing preferences and options with the patient. The recommendations for weight optimization (Table 2) are intended as strategies that will ultimately be selected and directed by the patient.

Guertin suggests that health professionals primarily focus on diet quality during counseling [19]. The traditional approach many clinicians use to advise people to limit food intake is based on the old maxim “calories in, calories out”. Pregnant individuals need to be thoughtful about portion size without restricting intake to lose weight.

Eating frequency is best considered in the context of an individual’s overall diet [6]. For example, the common recommendation in pregnancy to eat small, frequent meals in the first trimester to avoid nausea and vomiting and in later pregnancy to avoid heartburn from gastric reflux as the growing uterus places upward pressure on the stomach [61] contradicts the warning about grazing behavior increasing with increased BMI. In the first trimester of pregnancy, when nausea and vomiting are common, particularly when there are long gaps between meals, pregnant people are unlikely to skip breakfast. Individuals using small, frequent meals need to be mindful of daily intake totals. 

Having nutritious foods that include fruits and vegetables available in the home is a first step in eating foods that are less likely to contain unnecessary calories [15,16]. Eating regular meals with average-sized portions is likely to assist in reducing first-trimester morning sickness and third-trimester gastro-esophageal reflux [61]. Although small, frequent meals are often recommended to reduce nausea and reflux, grazing is associated with higher intake that may exceed energy needs [18]. Particular food regimens that are high in fruits and vegetables and low in saturated fats, including the Mediterranean diet, the DASH diet, low-glycemic index diets, and vegetarian diets, can provide nutrition for the pregnancy person and the fetus while avoiding calorie-dense foods that may cause excessive weight gain. Increasing dairy product intake is often recommended during antenatal care as a strategy to increase calcium intake to provide for growing fetal bones and prevent maternal bone loss. Pregnant people can be advised to use low-fat milk and dairy products, which will still contain the amounts of protein and calcium needed for pregnancy [61]. The use of dairy products within an energy-conscious diet can be advised with low concern for increasing excess gestational weight gain.

Physical activity, in addition to its cardiac benefits and its association with higher vaginal birth rates, increases non-insulin glucose uptake in muscles bypassing the physiologic insulin resistance of pregnancy and lowering the risk for gestational diabetes [62]. Not all pregnant people enjoy physical activity, and some have physical limitations, such as the low back pain of pregnancy that prevents activity. It is therefore important to remember that diet-based interventions in pregnancy are the most effective methods of optimizing weight gain and can limit gestational weight gain without additional physical activity [63]. Adequate sleep additionally lowers insulin resistance [64,65] and reduces opportunities for night-time snacking. Pregnant people should be cautioned not to decrease sleep in order to have more time for exercise. In one study, short sleep duration was significantly independently associated with higher body mass index (*p* < 0.001), body weight (*p* < 0.01), and waist circumference (*p* < 0.001). Fasting insulin levels were positively associated with sleep duration, and adiponectin levels were negatively associated. In total, 6 to 7 h of sleep in 24 h in this study was associated with the lowest obesity measures [64]. 

Acceptance of the body’s ability to signal hunger and satiety intuitively and accurately while denying the need for nutrition and weight gain counseling in pregnancy must be considered against the evidence that multiple factors change usual appetite signaling in those with obesity. Higher levels of cortisol and inflammatory cytokines in some individuals with obesity dysregulate the gut-brain axis with eventual blunting of central neural feedback networks, particularly gut-secreted incretins [65]. Intuitive eating additionally ignores growing recognition that nutrients and chemical signals produced by the gut microbiome are altered in people with obesity and change brain perceptions of hunger cues [66]. The principles of the Health at Every Size^®^ program are as follows: intuitive eating, self-acceptance, and using healthy eating and activity behaviors rather than losing weight [67] are consistent with the weight optimization guidance needed for antenatal care, yet many individuals will need more directive intake and activity guidance to avoid excessive gestational weight gain.

Excessive gestational weight gain has been a target for behavior change for several decades. A 2010 review of programs to limit gestational weight gain found that the programs included nutrition counseling but relied mainly on portion control and recommending increases in physical activity as weight management behaviors [68]. No program summaries contained specific nutrition pattern recommendations [68]. Food availability and eating patterns have changed over two decades. This review includes specific eating patterns, such as the Mediterranean diet and low-glycemic index diets that can be recommended during pregnancy. Other behaviors, such as improved sleep, mindful eating, and intuitive eating, have not been included in other synthesized reviews.

A recent scoping review of behavior change methods used during prenatal care examined programs that addressed alcohol use, smoking, physical activity, nutrition, and weight gain [69]. The most numerous types of behavior change programs reviewed were related to prenatal weight gain. The reviewers examined the following potential mechanisms for behavior change: empowerment, skills, competencies, innovation in the program, reflexive thinking by the patient, social support, supporting self-efficacy, and shared decision-making. All successful behavior change programs included education for knowledge gain or interactive education through health care provider support [69]. The eating patterns and behaviors detailed in this review can all be integrated into one-on-one provider-patient counseling or used in group health education.

## 5. Strengths and Limitations of Review

This scoping review was limited by the fact that little research is performed during pregnancy; therefore, evidence-based interventions studied outside of pregnancy that could be safely used during pregnancy were sought and summarized here. The magnitude of weight gain management research performed outside of pregnancy confounded study selection; however, nine evidence-based weight optimization strategies were identified (Table 2). A strength of this study is that eating patterns and behaviors used to manage weight outside of pregnancy were reviewed for consistency with recommendations by dieticians and safe use during pregnancy. This review synthesizes pregnancy-safe eating patterns and behaviors into a list that clinicians can use during the initial prenatal exam and subsequent antenatal care. 

## 6. Conclusions and Recommendations

Clinicians who provide antenatal care have an ethical obligation to discuss gestational weight gain with their patients along with the complications associated with excessive gestational weight gain. There is a balance to be practiced between supporting evidence-based weight management strategies and using weight-biased language or recommendations that might trigger disordered eating. Health care systems do not routinely incorporate nutrition counseling for healthy women during pregnancy by registered dieticians. Clinicians can recommend the evidence-based behaviors and eating patterns listed in Table 1 and Table 2 at the first prenatal education visit or later in care if weight gain does not meet target recommendations. This review provides clinicians with strategies for managing gestational weight gain that are consistent with national guidelines from dietetic associations.

More research is needed on strategies for managing gestational weight gain. Future research will be needed to investigate specifically which eating patterns and behaviors are most effective in optimizing gestational gain. For example, is eating a low-glycemic index diet more effective in controlling gestational weight gain than a usual Western diet with strict portion control? More research is also needed on the eating and sleep patterns of pregnant workers who work evening and night shifts and might have disordered eating and sleep patterns.

This systematic review updates the research literature related to eating patterns and behaviors that can be used safely during pregnancy to limit excessive weight gain. These strategies include meal planning, portion control, mindful eating, intuitive eating, eating patterns such as the Mediterranean, low-glycemic index, and DASH diets, intermittent self-weighing, and adequate sleep. The evidence-based strategies outlined in this scoping review can assist patients in limiting excessive gestational weight gain.

## Figures and Tables

**Table 1:** Foods and servings common to the Mediterranean diet, low-glycemic index diets, and the dietary approaches to stop hypertension diet.

FOODS	SERVINGS
Fruits	With every meal
Vegetables	With every meal
Whole grain cereals (wheat, rice, oatmeal, couscous, quinoa)	1–2 servings with every meal
Legumes (peas, pulses, beans, tofu products)	At least twice a week
Eggs	2–4 times a week
Dairy foods (milk, cheese, yogurt)	Twice a day, preferably low fat
Nuts, seeds, olives	1–2 servings per day
Potatoes, yams	2–3 times per week
Fish, seafood	At least twice a week
White meats (chicken, turkey)	2 servings per week
Red meats (beef, pork, organ meats)	1–2 times per week
Processed meats (lunch meat, sausages, deli meats)	Less than weekly
Plant oils (olive, sesame, peanut, canola)	Use for cooking in every cooked meal. Avoid saturated fats.
Sugar sweetened foods including sweetened sodas	1–2 times per week

**Table 2:** Evidence-based behaviors and eating patterns associated with weight management that are safe for use in pregnancy.

Planning contents and timing of meals
Portion control without intended weight loss
Mediterranean diet; DASH diet; low-glycemic diet; increased fruits, vegetables, fiber, and dairy in diet along with portion awareness
Increased physical activity
Adequate nighttime sleep
Mindfulness approach to eating
Intuitive eating
Self-weighing

## Data Availability

The data presented in this study are available on request from the corresponding author.

## Appendix A. Research Evidence Summaries for Eating Patterns and Weight Management Behavior Changes to Limit Gestational Weight Gain

**Table:**

Author, Year	N-Years Data Collected-Location	Study Type	Population Characteristics	Evidence-Based Weight Management Strategies	Results
Alshurafa et al., 2021 [51]	N = 16 (n = 8 with Obesity, n = 8 without); 2020 Chicago, USA	Prospective Cohort	Adults Age range: 18–65 BMI ≥ 18.5 kg/m^2^	Eating Behavior: Number of bites, eating speed, and time of day of eating episode and energy intake.	There were no significant interactions between obesity and number of bites, eating speed, or time of day (*p* > 0.05). Greater number of bites and reduced eating speed were significantly associated with higher energy intake in participants without obesity. No significant interactions between obesity, eating behaviors, or time of day with energy intake. Eating slowly associated with increased caloric intake-Mean (SE) BMI was lower among those that had faster versus slower eating episodes (23.50 [0.99] vs. 27.98 [2.42] kg/m^2^, respectively). Additionally, mean [SE] energy intake was lower in faster eating episodes (379.60 [59.56] kcal) than slower ones (632.80 [110.53] kcal), while eating duration was significantly shorter among the fastest eating episodes (46 [3.92] min) than the slowest (40.55 [34.31] min). A higher percentage of meals within the slowest eating episodes (80%) were consumed during screen time (i.e., watching TV, laptop, or phone) compared with the fastest eating episodes (30%). Number of bites and eating speed are potential eating behavior targets to prevent excessive energy intake.
Annesi, 2017 [70]	107 (53 experimental, 54 control); Southeast USA	RCT	Women with obesity Age 48.6 ± 7.1 years BMI 35.4 ± 3.3 kg/m^2^ Ethnicity 83% White, 11% African-American, 6% other Annual Household Income 11% < $25,000; 38% $25–$49,999; 40% $50–$99,999; 27% ≥$100,000	Social cognitive theory-based weight-management treatment group for 56 weeks Six 45 min coaching sessions over 6 months with wellness leader focused on self-regulatory skills and goal setting. Self-monitoring: Tracking intake. Group Sessions: Focused on self-regulatory skills; increased fruit, and vegetable intake; MyPlate, diet; assigned individualized energy intakes. Review of a written manual and phone support group 24 weeks. Self-directed review of LEARN (lifestyle, exercise, attitudes, relationships, nutrition) manual–12 lessons. 15 min phone call reviewing contents following lessons with wellness leader. Suggestion to limit energy intake to 1200 kcals/day and record dietary intake.	The experimental treatment was associated with significantly more favorable changes across variables. Over 6, 12, and 24 months, body satisfaction change mediated relationships between treatment type and changes in each of the psychological predictors of healthier eating (mood, self-efficacy, self-regulation).
Annesi, 2021 [71]	N = 97; Southeast USA	Prospective Cohort	Sedentary women with obesity Age M = 47.2 years BMI M = 34.8 kg/m^2^ (SD = 3.1) Ethnicities: 74% White, 22% Black, and 4% of other groups. Majority of household incomes: $50,000–$100,000/year.	Self-Regulatory Skills: Cognitive-behavioral weight-loss treatment over 1 year that emphasized building self-regulatory skills to maintain exercise in advance of transferring those skills to controlled eating.	Significant increase in exercise (metabolic equivalents per week or leisure score index [LSI]), and significant improvements in mood, self-regulation for exercise, and self-regulation for eating from baseline to months 6, 12, and 24. There were 5.9%, 5.8%, and 5.8% reductions in weight, respectively. Completion of 15–20 LSI did not significantly differ from greater amounts on associated weight losses except for the rare occurrence of ≥30 LSI over the full 24-month study period.
Cloutier-Bergeron et al., 2019 [67]	N = 210; 2010–2011; Montreal, CA, Canada	Retrospective Case-Control	Adults Age range: 21–83 years (M = 51.26, SD = 11.17)	Non-diet interventions: Choisir de maigrir (Choose to Lose Weight) is a non-diet intervention that is part of the HAES^®^ movement promoting intuitive eating, self-acceptance, and healthy behaviors rather than focusing on losing weight. Individualized Approach: Categorized into types of responders, which were then profiled on sociodemographic, weight, health, lifestyle, psychological and eating variables.	Categories: Non-responders (14.67%), moderate improvement with low maintenance responders (49.89%), moderate improvement with high maintenance responders (29.28%) and high functioning partial responders (6.56%). Some participants might experience too much psychological distress or disordered eating to actively take part in the non-diet intervention in order to fully benefit from it. Significant differences between all types of responders with medium to large effect sizes on depressive symptoms, self-esteem, and disinhibited eating (*p* < 0.001; η2 = 0.23, 0.30 and 0.16, respectively). Fewer differences were found on sociodemographic, lifestyle, health, and weight variables. Overall, non-responders (14.67%) had a distinctive profile compared to the other groups by consistently expressing poorer psychological functioning, less adapted eating behaviors and more frequently reaching the clinical cutoff for severe depression (*p* = 0.001).
Creasy et al., 2021 [42]	N = 84 2009–2012; University of Colorado-Anschutz medical campus, USA	Observational-cross-sectional	Weight loss maintainers (WLM): n = 28, maintaining ≥13.6 kg weight loss for ≥1 year, BMI 23.6 ± 2.3 kg/m^2^ Controls without obesity: n = 30, BMI similar to current BMI of WLM, BMI 22.8 ± 1.9 kg/m^2^ Controls with overweight/obesity: n = 26, BMI similar to pre-weight loss BMI of WLM, 33.6 ± 5.1 kg/m^2^	Sleep duration, sedentary time (SED), light-intensity PA (LPA), moderate-to-vigorous intensity PA (MVPA), steps.	Weight loss maintainers engaged in ≥60 min of MVPA on 73% of days, significantly more than controls with obesity (36%, *p* < 0.001) and similar to controls without obesity (59%, *p* = 0.10). Weight loss maintainers accumulated more MVPA in the morning (i.e., within 3 h of waking) compared to both controls with and without obesity (*p* < 0.01). Weight loss maintainers engaged in significantly more MVPA accumulated in bouts ≥10 min (*p* < 0.05). Weight loss maintainers engaged in more MVPA accumulated in bouts of ≥60 min compared to controls without obesity and OC (*p* < 0.05).
Denny et al., 2013 [52]	N = 2287; 1998–1999, 2008–2009; MN, USA	Cross-sectional analysis	1030 Males (45.2%) and 1257 Females (54.9%) Mean age 25.3 years ± 1.7 BMI groups: Normal: 442 male, 630 female Overweight: 357 male, 297 female Obese: 210 male, 305 female 48.4% White, 18.6% Black/African American, 5.9% Hispanic/Latino, 19.6% Asian American, 3.3% Native American/American Indian, and 4.3% mixed/other Socioeconomic Status: low 18%, low-middle 19%, middle 26%, high-middle 23%, and high 14%	Intuitive Eating: Participants were asked to indicate how strongly they agreed with the following two statements: “I trust my body to tell me how much to eat” and “I stop eating when I am full” using a four-point Likert scale (test–retest r = 0.65 [first question], r = 0.62 [second question])	Intuitive eating was inversely associated with BMI in both genders. 75.1% of women in the normal weight and underweight category indicated that they trusted their body to tell them how much to eat as compared to only 47.5% of those in the obese category (*p* < 0.01). Those reporting that they trust their bodies to tell them how much to eat had significantly lower odds for all disordered eating behaviors compared to those who reported that they did not trust their body.
Downs et al., 2021 [53]	N = 30; 2020; PA, USA	RCT	Control group-pregnant women with overweight (n = 16; 52%) Intervention group-obesity (n = 15; 48%) Age M: 29.6 (SD 4.1) At enrollment Gestational age M: 10.2 weeks (SD1.7) Weight M: 89.9 kg (SD 20.2) Pre-Pregnancy Weight M: 88.9 kg (SD 21.0) BMI M: 32.6 (SD 7.2) Racial Profile: Caucasian/white (n = 30; 97%), Asian (n = 1; 3%)	Weight Monitoring: Weekly evidence-based education/counseling with a dietician, gestational weight gain monitored weekly; women within weight goals continued with education while women exceeding goals received more intensive dosages.	Intervention group achieved 21% lower gestational weight than control group while having adequate prenatal weight gain.
Ferrara et al., 2020 [48]	N = 398 (200 intervention,198 usual care); 2014–2017; Kaiser Permanente; CA, USA	RCT	Women carrying singleton pregnancies Gestation: 8–15 weeks Pre-pregnancy BMI: 25.0–40.0 kg/m^2^ Age: >18 years	Behavior changes for weight management (e.g., daily self-weighing), Healthy eating (e.g., setting goals for eating healthy foods in appropriate portion sizes, total caloric intake, and calories from fat), Physical activity (e.g., 150 min per week of moderate-intensity to vigorous-intensity physical activity) Stress management Telehealth intervention delivered by dietitians using motivational interviewing techniques Approach to behavior change based on social cognitive theory and the transtheoretical model of change.	Compared with usual care, women in the lifestyle intervention had reduced weekly rate of gestational weight gain (mean 0.26 kg per week [SD 0.5] vs. 0.32 kg per week [0.13]; mean between-group difference of −0.07 kg per week, (95% CI −0.09 to −0.04). No between-group differences in perinatal complications were observed.
Greene et al., 2016 [72]	N = 7144 patient data, 2010–2012; 20 clinician interviews in 2013; Washington, DC, USA	Mixed Methods	Patients complete the 13-item patient activation measure (PAM) (n = 7144). PAM measures uptake of health behaviors recommended by clinicians. Top performing clinicians (n = 10) Bottom performing clinicians (n = 10)	Clinician Approach Strategies: Emphasizing patient ownership; partnering with patients; identifying small steps; scheduling frequent follow-up visits to cheer successes, problem solve, or both; and showing caring and concern for patients	Clinicians whose patients had relatively large PAM activation increases reported using 5 key strategies to support patient behavior change (mean = 3.9 strategies).
Guertin et al., 2023 [19]	N = 234 women; 2018–2019; Ottawa, Ontario	Prospective Cohort	Woman Age range: 17–72 (M = 30; SD = 11.31) BMI: Underweight (≤18.49; 2.5%) Normal weight (18.50–24.99; 57.9%) Overweight (25–29.99; 26.0%) Obese (≥30; 13.6%) Non-Hispanic White or European-American n = 188 (77.7%)	Planning and Self-monitoring: Planning and Self-Monitoring the Quality and Quantity Scale measured engagement in planning and self-monitoring strategies for the quality (i.e., nutrient intake) and quantity (i.e., calories and portion sizes) of their eating behaviors.	Planning and self-monitoring quality were significantly positively associated with healthy eating. Planning quality was significantly negatively associated with unhealthy eating, bulimic symptoms. Self-monitoring quality was non-significantly associated with unhealthy eating but significantly positively associated with bulimic symptoms. Planning and self-monitoring of calories and portion sizes were non-significantly associated with healthy and unhealthy eating behaviors and were significantly positively associated with bulimic symptoms. Planning portions was significantly positively associated with healthy eating. Healthy eating was significantly positively associated with life satisfaction. Unhealthy eating and bulimic symptoms were significantly negatively associated with life satisfaction at Time 3 in the study.
Harden et al., 2014 [73]	N = 137 pregnant women; 2013; Canada and United States	Randomized assignment, Mix Methods	Women Age: 21.9 years (+4.84 years) Racial identities: Caucasian (61.5%), black (23%), multiracial (7.6%), and unreported (7.6%) Average weight at baseline: 96.77 ± 13.97 kg Average BMI of 37.64 kg/m^2^ (+6.56) Gestation at recruitment: 11 weeks, 5 days (+2.7 days)	Group-Based Lifestyle Sessions (GBLS): Group visits were planned for one hour and included safe group exercises, nutrition education and demonstrations, and group-based activities (e.g., group goal setting)	After the 6-month intervention, the participants in the intervention condition had limited GWG at every time point, compared to those in the control condition: week 4 (0.66 ± 1.39 kgs. Versus 1.22 ± 2.57 kgs); week 8 (0.71 ± 1.72 kgs. Versus 3.12 ± 2.85 kgs); week 12 (2.12 ± 3.53 kgs. Versus 5.96 ± 3.81 kgs.); week 16 (5.22 ± 3.58 kgs. Versus 7.50 ± 3.58 kgs); week 20 (5.29 ± 5.33 kgs. Versus 8.64 ± 3.88 kgs). Only the differences in weight gain at 20 weeks were statistically significant (F(1,15) = 11.87, *p* < 0.01).
Jakicic et al., 2015 [74]	N = 195; 2003–2006; Pittsburgh, PA, USA	Randomized, controlled clinical trial of intervention	Adults Age, 43.2 ± 8.6 yr; BMI: 33.0 ± 3.4 kg·m^−2^	Three intervention groups received energy-restricted diet and physical activity prescriptions. Intervention groups also received time-based delivery of additional support interventions (phone calls about overcoming barriers, supervised physical activity, exercise campaigns) that were packaged into three programs (1) standard behavioral weight loss program, (2) ADOPT, and (3) MAINTAIN, over the course of a n 18-month weight management physical activity program.	Providing additional strategies over time improves fitness when compared to strategies given at the beginning to a standard program and the standard program alone. No significant between-group differences in weight loss from 0 to 6 months. Those who had additional strategies at predetermined times over the entire intervention period lost significantly more weight from 0 to 12 months compared with participants that had additional strategies only at the initiation of intervention (*p* = 0.0408), with a trend for greater weight loss when compared with standard behavioral weight loss program participants (*p* = 0.0558), with no significant difference between standard and early additional strategy intervention participants (*p* = 0.8850).
Knol, et al., 2021 [49]	N = 18; 2018–2019; AL, US	Quasi-Experimental	Pre-menopausal women Age range: 25–50 years BMI: 25–40 kg/m^2^	Mindful Eating Intervention: 8-week curriculum. Mindfulness techniques to develop an awareness of hunger and satiety cues, emotional states associated with eating, and external triggers to eat.	Measures: Perceived Stress Scale (PSS), Mindful Eating Questionnaire (MEQ), Eating and Appraisal Due to Emotions and Stress Questionnaire (EADES), BMI, waist circumference, blood pressure, and serum hydrophilic (H-AOX) and lipophilic (L-AOX) antioxidant capacity. PSS scores improved slightly. MEQ and stress related eating scores improved significantly (*p* = 0.001). Body weight (*p* = 0.02), waist circumference (*p* < 0.001), systolic blood pressure (*p* = 0.05), H-AOX (*p* = 0.02), and L-AOX (*p* < 0.001) all improved significantly.
Konsor et al., 2021 [16]	N = 196; 2014–2016; Chicago, USA	Secondary data analysis of a parent study	Adults Female n = 164 (83.7%) Age M = 44.38 years Ethnicity: American Indian/Alaskan Native n = 2 (1.0%) Asian n = 6 (3.1%) Black or African American n = 90 (45.9%) Native Hawaiian or Pacific Islander n = 1 (0.5%) Caucasian n = 70 (35.7%) Multi-Ethnic n = 15 (7.7%) Other/unable to choose n = 12 (6.1%)	Food planning Home food environment History of weight loss attempt(s)	Meal planning and grocery list use did not explain the relationship between a weight loss attempt and obesogenic foods or fruits/vegetables in the home (*p* > 0.05). In participants with a BMI of >25, >28, and >30, a weight loss attempt was associated with fewer obesogenic foods. In contrast, mediation analyses with planning behaviors and associations with fruit and vegetables in the home were nonsignificant (*p* > 0.05).
Latner et al., 2013 [75]	N = 67; USA	Observational-correlational	Adults Age M: 42.21 years (SD 15.07) BMI: 26.63 kg/m^2^ (SD 4.72) Baseline BMI: 30.65 kg/m^2^ (SD 4.97) Racial Profile: 94% were Caucasian	Self-efficacy to control eating behavior in various contexts and coping with high-risk eating situations (ability to generate a plan to limit or stop overeating in situations) were measured by survey. Participants kept a 7-day eating and activity control lapse diary.	Those who maintained <90% of their weight loss, had poorer self-efficacy, poorer coping, greater lapse frequency, and greater perceived lapse severity, than those who maintained at least 90% of their weight loss. Assessing self-efficacy, coping, and lapse perceptions prior to treatment may identify potential problem areas to target in weight maintenance programs.
Park et al., 2016 [76]	N = 50; Chicago, USA	Randomized, 2-arm, crossover	Woman Healthy Weight (n = 25) 26 ± 8 years old with mean BMI of 22 ± 2 (kg/m^2^) Obese (n = 25) 36 ± 13 years old with mean BMI of 33 ± 3 (kg/m^2^)	Gum chewing as a strategy to enhance lunch satiety and reduce afternoon snack intake.	GUM compared to Control resulted in significant suppression of hunger, desire to eat, and prospective consumption (*p* < 0.05). Total snack energy intake was reduced 9.3% by GUM, but not significantly different from Control (*p* = 0.08). However, overall carbohydrate intake was reduced by GUM (*p* = 0.03). Metabolic responses did not differ between experimental conditions.
Pearl et al., 2021 [77]	N = 13,996; 2020; UK, AUS, France, Germany, USA, Canada.	Cross-sectional survey	Adults enrolled in WW (formerly Weight Watchers) for a minimum of 3 months Age: >18 >90% of participants were female and white, Age range: 47 to 57 years >80% BMIs fell within the overweight or obesity categories Average BMIs: 29 to 31 kg/m^2^.	Health Message Dissemination: Health professionals must consider the potential for their messages about weight to be internalized in a negative way, and for internalization’s potential ironic effects on the very weight management behaviors and outcomes that the messages are meant to encourage.	Modified Weight Bias Internalization Scale (WBIS-M) scores were associated with greater weight gain in the past year. Participants with higher WBIS-M scores also reported poorer mental and physical HRQOL, less eating and physical activity self-efficacy, greater engagement in eating as a coping strategy, more avoidance of going to the gym, poorer body image, and greater perceived stress.
Porter et al., 2022 [78]	N = 210; 2016–2019; Greenville County, SC, USA	Observational-longitudinal	Patients referred by health care provider. Age: >18 Physically inactive (<150 min/week of moderate intensity aerobic activity) and/or Have a chronic condition (overweight/obesity, hypertension, type 2 diabetes, stable heart disease)	Clinic-to-community model for group physical activity program. Small group-level with ≤7 patients per group, 12-week duration with 60-min sessions twice a week. Exercise protocol conducted by certified EIMG^®^ Pros. Exercise prescription tailored to participant condition as needed. The training sessions guided by the social-cognitive theory following the principles that training is progressive, includes full body movements, and provides active education incorporated into training.	Exercise is Medicine Greenville (EIMG^®^) program participation paired with usual care was associated with a modest but statistically significant decrease in body weight and systolic blood pressure, with no significant decrease in resting heart rate or diastolic blood pressure.
Rolls et al., 2017 [22]	N = 186; 2012–2015; PA, USA	RCT	Women with obesity (81%) or overweight (19%) Age range: 20–65 years BMI range: 28–45 kg/m^2^	Portion control using tools (food scales, pre-portioned foods, single-serving main dishes).	Compared to control group that was instructed to eat less food while making healthy choices, the pre-portioned foods group initially lost weight at a greater rate and then regained at a greater rate. No significant weight loss difference between groups at months 6 and 12.
Roe & Rolls, 2020 [79]	N = 186; PA, USA	Secondary data analysis–See “Rolls et al., 2017”	Women with obesity (81%) or overweight (19%) Age range: 20–65 years BMI range: 28–45 kg/m^2^	Strategies in managing intake of self-identified “problem foods” (energy-dense desserts and snacks; low-fiber-carbohydrate-rich foods). Limiting strategies: -Reducing portions of intake -Substituting lower-calorie versions. Avoidance strategies: -Not keeping them available -Not buying them -Not eating them Other strategies: -Eating small amounts of problem foods as a treat or reward. -Decreasing frequency of intake without portion-control when consuming -Reliance on social support to help avoid eating problem foods	The strategy of limiting portions of problem foods was strongly related to the trajectory of weight loss in all intervention groups. Women who reported more frequently limiting portions of their problem foods had a greater rate of weight loss than those who used this strategy less frequently [F(2,186) = 11.23, *p* < 0.0001]. Those who frequently substituting lower-calorie versions of their problem foods for higher-calorie versions had a greater rate of weight loss than participants in this group who used this strategy infrequently. Significantly lower rates of weight loss in those who reported a higher use of eating problem foods less often but not worrying about the amount and (F(2,1010) = 6.76, *p* = 0.001) and in those who more frequently relied on social support to avoid eating problem foods (F(2,1023) = 5.55, *p* = 0.004). Three avoidance strategies were reported to be commonly used, after accounting for the other strategies none of them were related to weight loss: avoiding keeping problem foods available [F(1,141) = 0.00, *p* = 0.97], avoiding buying them [F(1,141) = 1.31, *p* = 0.25], or avoiding eating them [F(1,142) = 1.50, *p* = 0.22].
Saarikko et al., 2021 [80]	N = 16 2019–2022	Qualitative-Descriptive	Pregnant and postpartum women who were overweight (n = 11) Public health nurses (n = 5)	Health technology Technology assisted self-monitoring Community resources and support Education and motivation factors	The use of health technology was an element of antenatal care that could be used to approach the subject of weight and weight management. Smart wearables could also support an evaluation of the women’s lifestyles. The opportunity category highlighted the lack of resources for support during perinatal care, especially after birth. Both groups felt that support from the family was the most important facilitating factor besides motivation. The women also expressed a conflict between pregnancy as an excuse to engage in unhealthy habits and pregnancy as a motivational period for a change in lifestyle.
Shieh et al., 2015 [81]	N = 108 2013 IN, USA	Cross-sectional analysis of survey data	Female (50%); Male (50%) Age range: 20–49 years Ethnicity: Caucasian 86 (79.6%); African American 9 (8.3%); Hispanic 5 (4.6%); Others 5 (4.6%) BMI: Underweight ≤ 18.49; 3 (2.8%) Normal weight 18.50–24.99; 41 (38.0%) Overweight 25–29.99; 44 (40.7%) Obese ≥ 30; 20 (18.5%)	Nutrition Self-Efficacy Scar, Physical Exercise Self-Efficacy Scale, the Healthy Eating Change Strategies Scale, and the Physical Activity Change Strategies Scale were used to measure efforts in: Nutrition behaviors (fruit/vegetable consumption, dinner cooking, and restaurant eating) and exercise. Self-regulatory behaviors, including self-monitoring, problem solving, affect control, goal setting, self-reinforcement, and relapse prevention. Nutrition and exercise self-efficacy (ability to overcome barriers to eating healthy foods or physical exercise)	Self-regulation showed direct association with fruit/vegetable consumption and exercise. Self-efficacy had direct association only with exercise. To improve fruit/vegetable consumption of a client or a population, health professionals could develop interventions to enhance self-regulation capabilities, such as self-monitoring or setting concrete behavior change goals. For exercise, both self-efficacy and self-regulation play a direct role in influencing exercise behavior. Interventions that increase self-confidence, self-regulation, or both are likely to improve exercise in a person or a population.
Shieh & Draucker, 2018 [82]	N = 13; IN, US	Qualitative, semi-structured interviews	Pregnant People Mean age: 29.5 years African American 10 (76.9%) White 2 (15.4%) Multiracial 1 (7.7%) Obese 9 (69.2%) Overweight 4 (30.8%) First Pregnancy: No 10 (76.9%) Yes 3 (23.1%)	Self-monitoring of eating, walking, and weight gain using a weight scale, a food measuring cup, a water bottle, a pedometer, and tracking intake of vegetables, fruit, protein/dairy, fluids.	Responses were mixed. Some felt enhanced sense of self-control, increased mindfulness, planning, and trying new foods. Others reported stress related to not being able to keep up with self-monitoring regime.
Simonson et al., 2020 [50]	N = 24; Pittsburgh, PA, USA	RCT	Adults Age range: 18–55 years BMI: 18.5 and <40.0 kg/m^2^	Mindful Eating Speed of Eating	Mindful and slow eating strategies did not significantly decrease energy intake or improve satiety over test of two meals in participants. No significant difference in satiety ratings. Trend towards a decrease in energy intake in the mindful eating condition compared with the control condition and a prevention of increased intake in the slowed pace of eating condition.
Unick et al., 2022 [41]	N = 60 women; 2019–2020; The Miriam Hospital, Rhode Island	RCT	Woman BMI: 25 to <40.0 kg/m^2^ Age range: 18–60	12-week yoga intervention performed twice a week Structurally equivalent control group (cooking/nutrition classes) Following a 3-month behavioral weight loss program.	Among those with high initial weight loss (>5%), the yoga intervention group lost significantly more weight (−9.0 kg vs. −6.7 kg) at 6 months and resulted in greater distress tolerance, mindfulness, and self-compassion and lower negative affect, compared to control group.
Washburn et al., 2021 [43]	N = 298; 2016–2017; KS, USA	RCT	Adults with overweight or obesity	Physical activity (Regardless of volume)	No evidence for an association between the volume of moderate-to-vigorous intensity exercise and weight regain across 12 months following clinically relevant weight loss. Exercise volumes lower than those currently recommended for weight loss maintenance, when completed in conjunction with a behavioral weight-maintenance intervention, may minimize weight regain over 12 months. Participants randomized to 150, 225, and 300 min of exercise completed 129 ± 30, 153 ± 49 and 179 ± 62 min per week of exercise (supervised + unsupervised), respectively. Mean weight loss at 3 months (9.5 ± 3.1 kg) was similar across randomized groups (*p* = 0.68). Weight change across 12 months was 1.1 ± 6.5 kg, 3.2 ± 5.7 kg, and 2.8 ± 6.9 kg in the 150, 225, and 300 min per week groups, respectively.
